# A successful model of road traffic injury surveillance in a developing country: process and lessons learnt

**DOI:** 10.1186/1471-2458-12-357

**Published:** 2012-05-16

**Authors:** Junaid Abdul Razzak, Muhammad Shahzad Shamim, Amber Mehmood, Syed Ameer Hussain, Mir Shabbar Ali, Rashid Jooma

**Affiliations:** 1 Department of Emergency Medicine, Aga Khan University, Karachi, Pakistan; 2 Department of Surgery, Aga Khan University, Karachi, Pakistan; 3 Road Traffic Injury Research and Prevention Centre, Karachi, Pakistan; 4 Department of Urban and Infrastructure Engineering, NED University of Engineering and Technology, Karachi, Pakistan

**Keywords:** Road traffic injury, Surveillance, Developing country

## Abstract

**Background:**

Road Traffic Injuries (RTIs) are one of the leading causes of death and disability worldwide with 90% of global mortality concentrated in the low and middle income countries. RTI surveillance is recommended to define the burden, identify high risk groups, plan intervention and monitor their impact. Despite its stated importance in the literature, very few examples of sustained surveillance systems are reported from low income countries. This paper shares the experience of setting up an urban RTI surveillance program in the emergency departments of five major hospitals in Karachi, Pakistan.

**Method:**

We describe the process of establishing a surveillance system including assembling a multi-institution research group, developing a data collection methodology, carrying out data collection and analysis and dissemination of information to the relevant stakeholders. In the absence of a road safety agency, the surveillance system required developing individual partnerships with industry, police, city government, media and many other stakeholders. Impact of the surveillance is demonstrated by some initiatives in the local trauma system and improvements in road design to effect hazard reduction.

**Conclusion:**

We demonstrated that a functional RTI surveillance program can be established, and effectively managed in a developing country, despite lack of infrastructure and limitation of resources. Data utilization in the absence of well defined road safety infrastructure within the government is a challenge. More effective actions are hampered by the limited capacity in the transport and health sectors to do in-depth analysis through road safety audits and trauma registries.

## Background

Road Traffic Injury (RTI) is one of the leading causes of death and disability worldwide. The Global Burden of Disease Study estimated that by 2030, RTI will become the third leading cause of lost disability-adjusted life years (DALYs) [1,2]. Low and middle income countries (LMIC) account for almost 90% of global mortality due to RTI.

Pakistan is 5th leading contributor to deaths due to RTIs worldwide with an estimated 41494 deaths per year [3]. Earlier work has shown injuries to be the second leading cause of disability, the eleventh cause of premature mortality, and the fifth cause of lost healthy life-years (HeaLY) [4,5]. The estimated RTI incidence in Pakistan as per its first national survey in 1997 is 15.1 per 1000 [5].

Improvement in data on road traffic injuries and deaths is considered a key for successful implementation and monitoring of road safety programs. [6] Globally, the world report on road traffic injuries, as well as recently released Global Status Report on Road Safety highlighted the need for setting up surveillance systems in LMICs [1,7]. In the Eastern Mediterranean region, standardizing definition and data collection methodology was identified as one of the recommendations for containing the epidemic of road traffic injuries in the region. [3] Similarly, earlier work in Pakistan has identified surveillance as a key to initiation and implementation of a successful injury prevention program [8,9].

Examples of real time, sustained surveillance systems are difficult to find in low income countries (LICs). This paper shares the experience of setting up an urban RTI surveillance program in the emergency departments of five major hospitals in Karachi, Pakistan. We describe the process of establishing a surveillance system including the assembling of a multi-institution research group, developing a data collection methodology, carrying out data analysis and dissemination of information to the relevant stakeholders.

## Methods

### Setting – the Karachi city

Karachi, the largest city of Pakistan is also the 14th largest metropolitan city of the world. With an estimated population of more than 16.2 million in 2007, the city is the financial, industrial and trade capital of Pakistan [10]. Karachi has large number of government and private sector hospitals. Prior to 2004, all trauma victims in the city required a visit to a government designated trauma hospital to obtain a document certifying patients’ evaluation by the medico-legal officer/police before care could be initiated. Following a change in the law, there is a growing trend to seek care at the private hospitals, though majority of trauma victims still continue to receive initial care at the three government trauma centers. There are many private ambulance services in the city most of which provide only taxi services in a stretcher. In the absence of outpatient health information management system, no medical records are kept for visits to the emergency departments at two of the three government hospitals. None of the hospitals have a functional trauma registry.

### Assembling a research team

Initially a core group of researchers interested in road safety got together. The group comprised of two health professionals, one civil engineer and a retired senior officer of police. Previous work on road safety carried out in Pakistan was reviewed and became the basis for establishment of a formal surveillance system for RTIs. The review included published research describing the ambulance data, medico-legal data, hospital based discharge data [11] and trauma victims coming to the government and private hospitals. [12] No formal need assessment was carried out as part of setting up of the surveillance system. There were three main objectives of the surveillance system: 

 a - to ascertain the burden of road traffic injuries presenting to major hospitals of the city,

b - to associated with road traffic and 

c - to identify possiblesolutions for reducing the burden of RTIs in the city.

**Figure 1:**
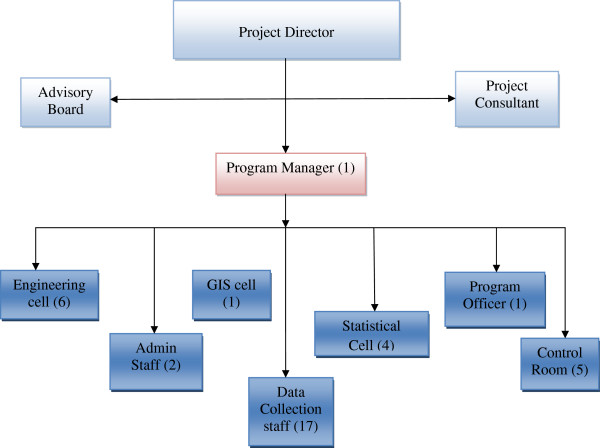
**Organizational structure of RTIRP. **Numbers in bracket indicate number of people working in given section.

The group developed the surveillance methodology and data collection form based on the available international guidelines [13]. A corporate organization agreed to financially support the project as part of their corporate social responsibility mandate. A program coordinator, with a background in safety was hired to run the project and a surveillance supervisor was hired for each of the five institutions. While the initial funding was through the corporate sector, the space for the data management team was provided by one of the medical institution. Approval from institutional research review board was obtained. An organizational structure was developed as shown in Figure 1.

### Data collection

The pro-forma (Appendix 1) consisted of about 20 data items, and included demographic information, contributory factors, details of injury mechanism and severity, vehicular information as well as means of transport and eventual outcome. The pro-forma was piloted and modifications were made prior to formal initiation of data collection. Data collectors were trained in administration of the surveillance instrument, medical data abstraction, ICD-9 CM coding and Injury scoring systems. Trauma patients presenting to the emergency department were screened for road traffic crash as the cause of the injury by the data collectors. All patients, irrespective of the severity of injury, were included in the surveillance. Data was collected from the victims themselves whenever feasible. In other cases, any eye witnesses, ambulance drivers, or relatives were interviewed. In cases where information was not available during patients’ treatment in the emergency department, data collectors visited patients in the hospital to interview patients or their family members and occasionally hospital’s inpatient record and/or police records were used when no other source of information was available to obtain the requisite information. Data quality was assured by the site supervisor based at each hospital as well as by the random checks done by the investigators and project coordinators. The surveillance system was simultaneously launched in all five hospitals in September 2006 with data collectors working 24 hours a day in three 8 hour shifts in the three major government hospital. For two private hospitals with Emergency Department records and a low trauma volume, one surveillance officer collected data on all patients seen during the 24 hour period. After about one year of surveillance, a system of following up patients admitted to the inpatient units was initiated to determine their outcomes. Also the system of recording information from medico-legal registers was started to obtain data on victims who were taken directly to the medico-legal department instead of bringing them to hospitals. This occurred usually when patient died at the scene of injury.

### Data analysis, geographical and root cause analysis

Frequency analysis was performed describing victims, vehicles and places. Geographical site of crash was determined as closely as possible. This sometimes required visits to the site of crash. Whenever possible, geographical co-ordinates were established on the satellite images of city of Karachi using the Geographic Information System (GIS). The satellite images were downloaded from Google Earth with geographical plotting within 50 meters of the site of event. For crash sites with high frequency of crashes, collaborating engineers from the NED Engineering University along with project staff visited the site and carried out a road safety audit for possible causative factors such as an awkward turning angle, open manholes, slopes, oil spillages etc.

### Data dissemination strategy

A number of steps were taken to use the information obtained from the surveillance project. Firstly, a report was generated from the collected data every three months and forwarded to multiple stakeholders to derive medical, engineering and law enforcement interventions. The stakeholders included the city government, traffic, highway and city police, citizen-police liaison committee, ambulance service providers, hospital administrators, ministry of health, corporate partners, and print media. Secondly, data was presented at specially convened meetings at regular intervals between the project team and the concerned officers and leadership of the municipal authority to discuss potential interventions. Thirdly, an annual conference was organized to share the findings with the general public and high level government functionaries such as Minister of Communication and Transportation. Finally, the results of road safety audits were shared in person by the project staff with the concerned authorities in the city government and the traffic police.

### Cost of running the surveillance and sustainability

The bulk of cost of the surveillance system included the salary of the staff, cost of computers/printers, phone/internet and printing of forms. These costs amounted to USD 45000 per year. About two third of the cost is for salaries of the data collectors. The cost of time spent by the senior researchers and the space is estimated to be another USD: 15000 per year.

## Results

### Impact of surveillance system

The surveillance system has led to an increase in focus among the police and civic agencies on the issue of road safety. The main finding of the surveillance was the high number of deaths and injuries compared to the numbers reported by police, the official source of road traffic injuries and deaths. Our study found that at best, only half of all road traffic injury deaths and only 2-3% of non-fatal injuries are reflected in the police records. This major difference assured that the surveillance received attention. (Table 1) There are two possible reasons for this difference: 

a-many patients were brought to the hospital from the site of crash before the arrival of police and

b-deaths in police record captures death either at the scene or soon after arrival to the emergency department but does not include patients followed up after admission to hospitals

The recommendation of actions to enhance road safety based on evidence derived from the injury surveillance has been a priority for the Project and will be detailed in a future publication. In the domain of health services, the hospital which received the largest number of injured, many of whom had head and neck injuries, has upgraded its neurosurgical facilities. Similarly, sharing of data regarding differences in outcomes of trauma care between the participating centers, led the management of one of the poorly performing hospitals to make efforts to improve their system of care. Having been sensitized by the results of the surveillance data, the City Government of Karachi made the decision to invest in a de-novo pre-hospital care system with a newly trained cadre of paramedics and a fleet of BLS Ambulances positioned at the sites with highest density of crashes reported by the surveillance project. The system is yet to become completely functional.

**Table 1:** Difference between police data and the surveillance data obtained through RTIPC

**Year**	**Police records**		**RTIPC surveillance**	
	Deaths	Injuries	Deaths	Injuries
2008	615	830	1185	32497
2009	510	606	1288	31051
2010	491	608	1227	30340

On the engineering side, the project identified 50 spots with highest risk frequency of road crashes. Safety audits were conducted on these spots by the engineering arm of research team and many of the recommendations of the group were adopted by the city government. These measures include development of overhead pedestrian bridges, reducing speed by introducing traffic calming devices, ensuring pedestrian safety through development of footpaths and street lights and coupling of bus stands with zebra crossings and others.

The surveillance project has been able to provide regular reports to the news media. As a result, several public awareness campaigns were launched by various partners including police on the need for helmet use, speed control and general awareness about road safety.

## Discussion

The project team has tried to use the information obtained from the surveillance to bring policy and practice change among various stakeholders. During the process, a lot of valuable lessons were learnt and are worth mentioning here.

It takes time and effort for getting a buy-in of data and converting data into actions. We initially encountered widespread disinterest and skepticism among various agencies responsible for road and traffic management. However over time, with the dissemination of our quarterly reports and ongoing dialogue, there is now greater confidence of these agencies in the data. They are also more willing to share the data generated from their own record keeping.

Lack of a lead agency for road safety results in dilution of responsibility for road safety and lack of ownership of the problem. This leads to poorer utilization of information available through surveillance.

Lack of pre-hospital and hospital based trauma care system at the city level led to challenges in finding users of the medical information. Currently, the hospitals involved represent the three different tiers of government: federal, provincial and city government, as well as multiple pre-hospital care providers and many private hospitals. Coordination between these varied stakeholders is difficult and discussing differences in outcome often leads to less willingness for institution to share their patient information.

We have learnt that even a robust surveillance system such as this is likely to miss minor injuries that were either never treated or were treated at a small hospital or clinic [13].

In the first three years of the project a private industry sponsor supported the project. Such a system is dependent upon the performance of the industry and therefore quite vulnerable to the changing economic scenario. A long-term stable source of funding is required for ensuring a stable surveillance system. Eventually, Government’s ownership and direct financial support of the surveillance system will be needed. In low income countries such as Pakistan, often the competing priorities, limited health and health research budgets and poor understanding of the role of injury surveillance are some of the reasons for lack of support.

The information obtained through surveillance system often required to be supplemented with additional information not possible in surveillance projects. The internal capacity within the health system or the civic agencies to gather the in-depth information is often lacking either due to capacity issues or lack of focus. Systems need to be developed to conduct road safety audits and a system of trauma registry needs to be developed to provide action oriented information for agencies in low income settings.

## Conclusion

This project provides an example of how a functioning road traffic injury surveillance system can be set up and sustained over time in a large urban setting in a low income country. Such a system required innovative partnerships and funding mechanisms. The actions on the surveillance data are difficult in the absence of a lead agency. We propose linking whenever possible the surveillance system with an active system of road safety audits and a system of trauma registry to strengthen the prevention and care aspects of the road traffic injury victims.

## Appendix 1

“Data collection form”

## Abbreviations

RTI: Road Traffic Injury; DALY: Disability-Adjusted Life Years; LMIC: Low and Middle Income Countries; HeaLY: Healthy Life-Years; LIC: Low Income Countries; GIS: Geographic Information System; BLS: Basic Life Support.

## Competing interests

This work was initially supported by a grant from Indus Motor Company Ltd, and the World Health Organization, Regional Office for the Eastern Mediterranean Region.

## Authors’ contributions

JAR was the primary author of the manuscript. AM, MSS, MAH, MAS and RJ provided critical review and final approval of this version. All authors read and approve the final manuscript.

## Authors’ information

**JAR** (MD, PhD, and FACEP) is the Associate Professor and Chair, Department of Emergency Medicine, Aga Khan University, Karachi, Pakistan.

**MSS** (MBBS, MRCS, FCPS) is Assistant Professor and Consultant Neurosurgeon in the Department of Surgery, Aga Khan University, Karachi, Pakistan.

**AM** (MBBS, FCPS), is Finklea Fellow for Injury Prevention and Research, and Assistant Professor in the Department of Emergency Medicine, Aga Khan University, Karachi, Pakistan.

**MAH** (BE) is the project coordinator for the Road Traffic Injury Research and Prevention Center.

**MSA** (PhD) is Professor and Chairman Department of Urban and Infrastructure Engineering at the NED University of Engineering and Technology Karachi Pakistan.

**RJ** (FRCS, FRCSEd-SN) is Professor of Neurosurgery in the Department of Surgery, Aga Khan University and Principal Investigator, Road Traffic Injury Research & Prevention Centre, JPMC, Karachi.

## Pre-publication history

The pre-publication history for this paper can be accessed here:

http://www.biomedcentral.com/1471-2458/12/357/prepub
